# Patient and proxies’ attitudes towards deferred consent in randomised trials of acute treatment for stroke: A qualitative survey

**DOI:** 10.1177/23969873211057421

**Published:** 2021-11-13

**Authors:** Noa van den Bos, Sophie A van den Berg, Catalina MM Caupain, Jeannette AJ Pols, Tessa van Middelaar, Vicky Chalos, Diederik WJ Dippel, Yvo BWEM Roos, Manon Kappelhof, Paul J Nederkoorn

**Affiliations:** 1 Department of Neurology, 26066 Amsterdam UMC, University of Amsterdam, Amsterdam, The Netherlands; 2 Department of Ethics, Law and Humanities, 26066 Amsterdam UMC, University of Amsterdam, Amsterdam, The Netherlands; 3 Department of Anthropology, University of Amsterdam, The Netherlands; 4 Department of Neurology, 6993 Erasmus University Medical Center, Rotterdam, The Netherlands; 5 Department of Radiology & Nuclear Medicine, 522567 Amsterdam University Medical Centers, University of Amsterdam, Amsterdam, The Netherlands

**Keywords:** Acute ischaemic stroke, deferred consent, endovascular treatment, informed consent

## Abstract

**Introduction:**

Deferral of consent for participation in a clinical study is a relatively novel procedure, in which informed consent is obtained after randomisation and study treatment. Deferred consent can be used in emergency situations, where small therapeutic time windows limit possibilities for patients to provide informed consent. We aimed to investigate patients’ or their proxies’ experiences and opinions regarding deferred consent in acute stroke randomised trials.

**Patients and methods:**

For this qualitative study, Dutch Collaboration for New Treatments of Acute Stroke (CONTRAST) trial participants were selected. Study participants were either patients or their proxies who provided consent and were selected with theoretical sampling based on patient characteristics. Semi-structured interviews were conducted face-to-face or by telephone. Themes and subthemes were iteratively defined.

**Results:**

Twenty of the 23 interviewed participants (16 patients and 7 proxies) considered deferred consent acceptable. The received study treatment and consent conversation were remembered by 18 participations, although the concept of randomisation and treatment comparison were generally not well understood. Sixteen participants felt capable of overseeing the decision to give deferred consent. Distress in the first days after stroke, lack of understanding and neurological deficits were reasons for feeling incapable of providing consent. Four participants would have preferred a different timing of the consent conversation, of whom two prior to treatment.

**Conclusion:**

Our study found that deferred consent was considered acceptable by most study participants who provided consent for acute stroke randomised trials. Though they felt capable, the recall and comprehension of consent were overall limited.

## Introduction

Since endovascular treatment (EVT) for acute ischaemic stroke due to large vessel occlusions is now standard of care, new clinical trials focus on further improving outcomes of EVT.^1^ Acquiring informed consent before randomisation or acute study treatment from patients – or proxies in case of decision incapacity – can be challenging in the acute setting of stroke.^2–5^ Neurological deficits or stress may impede patients’ or proxies’ understanding and capability to provide consent.^2^ This could lead to selection bias, if patients with severe neurological deficits in trials are less likely to be included in trials.^2–4,6,7^ By delaying treatment, asking consent beforehand might actually harm patients^5,8^: in the first six hours from stroke onset, chances of good outcome decrease by 6%/hour for EVT-eligible patients.^4,9^

Deferral of consent may address these issues. In this relatively novel approach, consent is asked after randomisation and study treatment.^2,7^ Several studies in other fields have used deferred consent and suggested that most participants found it acceptable.^5,10–13^

However, deferring consent may raise ethical concerns. Do the benefits of fast treatment and unbiased study inclusion outweigh the concerns of deferring consent? Several studies have assessed physicians’ opinions or outlined the ethical principles at play.^2,14–17^ Deferred consent for medical research is approved in many world regions, including Europe, North America and Australia, and is described as a valid option in the Declaration of Helsinki and the International Ethical Guidelines for Health-related Research Involving Humans.

However, data about patients' own knowledge, opinions and emotions concerning deferred consent are scarce.^2^ In the current qualitative study, we investigated patients’ and proxies’ experiences with deferred consent for participation in the Collaboration for New Treatments of Acute Stroke (CONTRAST) trials (Table 1).^14,18–20^

**Table 1.** Collaboration for new treatments of acute stroke trials on treatments for acute ischaemic stroke.

Trial acronym	Full name	Treatment comparison	ISRCTN Registry number
MR ASAP	Multicentre Randomised trial of Acute Stroke treatment in the Ambulance with a nitroglycerin Patch	Prehospital treatment with transdermal nitroglycerin 5mg/day vs. standard care in suspected stroke	ISRCTN99503308
MR CLEAN-LATE	Multicenter Randomised CLinical trial of Endovascular treatment for Acute ischemic stroke in the Netherlands for late arrivals	EVT vs. best medical treatment for patients presenting between 6 and 24 hours after stroke onset or last seen well	ISRCTN19922220
MR CLEAN-MED	Multicenter Randomised CLinical trial of Endovascular treatment for Acute ischemic stroke in the Netherlands: the effect of periprocedural MEDication: heparin, antiplatelet agents, both or neither	Acetylsalicylic acid, unfractionated heparin, both, or none during EVT	ISRCTN76741621
MR CLEAN-NO IV	Multicenter Randomised CLinical trial of Endovascular treatment for Acute ischemic stroke in the Netherlands: intravenous treatment followed by intra-arterial treatment versus direct intra-arterial treatment for acute ischaemic stroke caused by a proximal intracranial occlusion	Direct EVT compared to intravenous alteplase followed by EVT	ISRCTN80619088

CONTRAST: collaboration for new treatments of acute stroke; EVT: endovascular treatment.

## Methods

### Setting and participants

This study explores patients’ and proxies’ views on and comprehension of deferred consent in the ongoing or recently completed CONTRAST consortium trials on acute treatments for acute ischaemic stroke (www.contrast-consortium.nl). Details on the four individual trials, one ambulance trial and three EVT trials can be found in Table 1 and were reported previously.^14,18–20^ Consent was asked as soon as possible after treatment but at least within three months, from patients or from their proxies if patients were incapable to give consent.

Trial participants were considered for the current study if they gave consent for the trial and permission to be approached for additional studies. Patients who gave consent for the trial themselves but had died or were severely aphasic were excluded. If a proxy gave consent for trial participation on the patients’ behalf, the proxy was included as a participant in the current study. Patients or proxies who could not be reached by telephone after three attempts were excluded. For each CONTRAST trial, ten potential participants were contacted during hospital admission or afterwards by telephone. We used theoretical sampling based on the following: patient age, sex, treatment centre, date of study treatment and stroke severity assessed with the National Institutes of Health Stroke Scale (NIHSS).^21^ Participants were asked to provide recorded verbal informed consent for this study at the beginning of the interview. Interviews were performed until data saturation was reached, that is, when no new (sub)themes arised.^22^ Ethical approval for all trials was obtained from the central medical ethical committee of the Erasmus Medical Center, Rotterdam and the research boards of each participating centre.

### Data collection and survey design

Between February 2020 and May 2020, one researcher (NvdB) performed in-depth, semi-structured interviews (online Appendix I) of approximately 40 min. Interviews were held at the homes of patients or proxies, at the hospital during admission or by telephone. If patients had mild aphasia, their partner or children were allowed to join the interview. All interviews were audiotaped. The interview guide (online Appendix I) was designed in cooperation with a qualitative research expert (AJP). Its questions were iteratively adjusted based on insights gained from the interviews (Online Appendix I). The interviewer was not involved in the CONTRAST trials or treatment of the patient.

### Coding and analysis

The interviews were transcribed verbatim and independently coded and analysed by two investigators (NvdB and CMMC), and subsequently compared and discussed. The codes were divided into themes and subthemes (Online Appendix III, Supplemental Table S1). Descriptive statistics were used to summarise participants’ replies and baseline characteristics. All shown citations are translated from Dutch to English.

## Results

**Figure 1.**
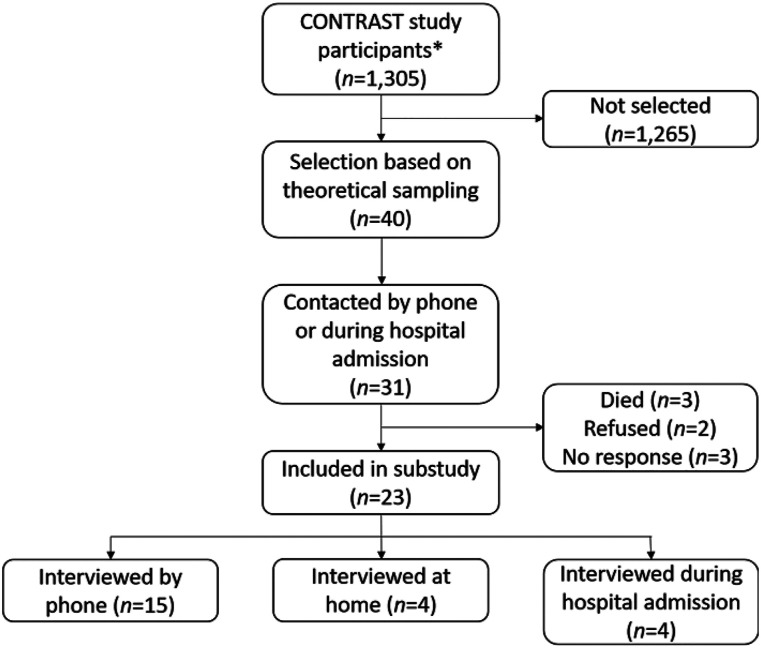
Inclusion flowchart. *Participants of MR CLEAN-NO IV, MR CLEAN-MED, MR CLEAN-LATE and MR ASAP. For three patients who had died, we could not include a proxy since consent was not given by a proxy. MR CLEAN, Multicenter Randomised CLinical trial of Endovascular treatment for Acute ischemic stroke in the Netherlands; MR ASAP, Multicentre Randomised trial of Acute Stroke treatment in the Ambulance with a nitroglycerin Patch.

At the time of our study, 1305 patients were enrolled in the CONTRAST trials (February 2020). Theoretical data saturation was reached after 23 interviews, with 16 patients and seven proxies between 3 February 2020 and 4 May 2020 (Figure 1, Table 2). Partners or children of patients participated in six interviews. Patient age ranged from 33–90 years (mean 68 ± 16), and baseline NIHSS from 2–29 (median 9; IQR 5–17). Median time between randomisation and consent was one day. Two patients had a relatively long delay (97 and 69 days, respectively). A summary of responses to yes/no questions can be found in Online Appendix II, Supplemental Figure S1 and Online Appendix III, Supplemental Table S6.

**Table 2.** Baseline patient and study characteristics.

	Patient characteristics				Study characteristics			
no	Sex	Age (years)	BL NIHSS	BL aphasia	Trial	Days between randomisation and consent	Days between consent and interview	Consent signed by
1	M	45	2	No	MR ASAP	5	45	Patient
2	F	47	2	No	MR ASAP	0	81	Patient
3	M	65	5	No	MR ASAP	1	33	Patient
4	M	73	9	No	MR ASAP	0	98	Son
5	F	84	3	Mild/mod.	MR ASAP	1	36	Patient
6	M	56	4	No	MR CLEAN-LATE	0	1	Patient
7	M	68	27	Severe	MR CLEAN-LATE	97	271	Daughter
8	F	83	7	No	MR CLEAN-LATE	2	162	Son
9	M	87	5	Mild/mod.	MR CLEAN-LATE	0	120	Partner
10	M	88	4	No	MR CLEAN-LATE	1	310	Son
11	F	45	23	No	MR CLEAN-MED	0	1	Patient
12	F	71	6	No	MR CLEAN-MED	1	69	Patient
13	F	74	16	Severe	MR CLEAN-MED	1	67	Patient
14	F	81	29	Mute	MR CLEAN-MED	0	73	Partner
15	M	82	9	Mute	MR CLEAN-MED	1	88	Patient
16	F	90	22	Mute	MR CLEAN-MED	0	57	Son
17	F	33	20	Mute	MR CLEAN-NO IV	1	83	Patient
18	F	55	13	No	MR CLEAN-NO IV	1	0	Patient
19	F	61	3	No	MR CLEAN-NO IV	0	141	Patient
20	M	63	9	Mute	MR CLEAN-NO IV	69	—	Patient
21	M	63	11	No	MR CLEAN-NO IV	1	269	Patient
22	M	66	5	No	MR CLEAN-NO IV	3	321	Patient
23	M	74	17	Mute	MR CLEAN-NO IV	2	281	Patient

BL: baseline; F: female; M: male; mod: moderate; MR CLEAN: Multicenter Randomised CLinical trial of Endovascular treatment for Acute ischemic stroke in the Netherlands; MR ASAP: Multicentre Randomised trial of Acute Stroke treatment in the Ambulance with a nitroglycerin Patch; NIHSS: National Institutes of Health Stroke Scale; No: patient number.

### Theme 1: Research and study methods

#### Comprehension of the trials

Nearly all participants remembered they participated in research and which stroke treatment they had received, although most of them thought participation only concerned follow-up monitoring. Of those who remembered the different treatment options (Supplemental Table S2, Q1), most had previous experience with medical research and had read the information forms. Lack of explanation of the study methods was mentioned for most trials. Most MR ASAP participants could recall the study methods and had often received information in the ambulance (Supplemental Table S2, Q2). Reasons for not comprehending study methods were (1) too many things happening at once, (2) rushed conversations or (3) distress in the first days after stroke:

*‘But, furthermore, I have not… It all happened and all in a panic at the beginning. And then a lot was said and, but well, that is all missing. I do not know all that’.* [P14]

Four patients and one proxy knew they had received treatment by randomisation (22%). Randomisation was considered strange by some, because they felt uneasy with receiving a treatment based on chance. A few participants were current or former healthcare workers; they understood the study methods (Supplemental Table S2, Q4 and Q5).

### Theme 2: Deferred consent procedure

#### Deferred consent conversation

Of 18 participants who remembered the deferred consent conversation, most only remembered specific actions like signing papers. Some could not remember the content or admitted they had forgotten (Supplemental Table S2, Q6). Insufficient time for the conversation, conversations held shortly after the acute treatment, use of medical jargon and language barriers reduced understanding. Explanation was satisfactory for most participants, although some had preferred more clarification of the complicated topics (Supplemental Table S2, Q7). Information forms were often appreciated, though not always read. Participants who read the papers understood the reasons for using deferred consent (Supplemental Table S2, Q8).

#### Reasons for consent

As reasons for giving consent, most participants expressed trust in their physician or the healthcare system (Supplemental Table S2, Q9). Others considered the expected research results important and felt that participating in research is part of being treated in a university hospital (Supplemental Table S2, Q10, Q11).

#### Feeling of capability

More than half of the participants (16/23) felt capable to provide consent at the time of the conversation (Supplemental Table S2, Q12). Patients with remaining stroke symptoms more often felt incapable to give consent:

*‘In my brain, I know everything. But I can’t express … I can’t express it. That was my problem. And it still is’.* [P23]

Presence of relatives during the consent conversation was often appreciated or desired and made patients feel more capable to decide about consent (Supplemental Table S2, Q14 and Q15). A few patients mentioned they had left the conversation to a family member and only signed themselves. Although these patients remembered very little, their family members often recalled detailed information about deferred consent and study methods (Supplemental Table S2, Q16). Two patients argued that physicians would always be more capable than patients or proxies, and hence should decide on their behalf on trial participation (Supplemental Table S2, Q17). Proxies considered providing consent to be their responsibility, and would not have wanted anyone else to decide – except for one proxy, who would have agreed with the physician or another family member providing consent (Supplemental Table S2, Q18).

#### Best timing

Participants were mostly satisfied with the timing of the consent conversation after treatment. Two proxies would have preferred to make the consent decision before randomisation and study treatment, though they would have given consent either way (Supplemental Table S2, Q19). Opinions on the best timing of the consent conversation varied from as soon as possible, in order to maintain their autonomy, to months after treatment, when everything had come to rest and they would have had time to think about the decision (Online Appendix III, Supplemental Table S). Twenty-two patients felt there was sufficient time for reflection (Supplemental Table S2, Q20). One patient thought any information given before treatment would not have been comprehended (Supplemental Table S2, Q21).

#### Acceptability of deferred consent

Almost all participants found deferred consent acceptable (20/23; 87%). They mentioned the necessity of fast stroke treatment, the distress and incapability to give consent in the emergency setting and trust in healthcare as justifications for deferral of consent. Two proxies did not state a clearly positive or negative opinion, and one proxy did not think deferred consent was acceptable:

*‘What’s done is done. Then you can’t really go back, kind of. […] If you know things in advance, you can say yes or no. And when it’s already done, that just makes me think “yes, oh, wait a minute”’’*[P8]

Three participants believed that consent should not be asked at all. Some found deferred consent more appropriate than asking consent before randomisation:

*‘Yes, that’s why guys, and uh, at that point, action is needed, and you don’t have time to explain for half an hour first, to explain something to, to depict how it all works. […] At such a moment, action is needed and we don’t have time to quibble over rules first’.* [P16]

Patients’ perceptions and feelings on deferred consent for trial participation are shown in Online Appendix III, Supplemental Table S5. A low additional burden or few study procedures were important for acceptability of deferred consent, though most participants mentioned that any change in these factors would probably not have changed their decision to participate (Supplemental Table S2, Q24 and Q25).

## Discussion

Most participants in this study reported acceptance of and a positive experience with deferred consent for continued participation in a randomised controlled trial of acute stroke treatment. Participants experienced difficulties in recalling and comprehending the content of the deferred consent conversation and study methods. However, most felt capable of providing consent when they did so. Patients with remaining stroke symptoms more often felt incapable to give consent. Opinions about the best timing for consent differed.

Our findings are generally in line with previous studies on both informed and deferred consent in acute stroke^6,23–25^ or other emergency settings.^10–12,16,17^ In 2008, Mangset et al.^23^ performed semi-structured interviews with stroke patients about their experience with informed consent before randomisation for a trial on thrombolysis. Patients who refused participation in the trial were also invited. Although this study does not concern deferred consent, the results were similar to ours regarding reasons for giving consent, and difficulties of remembering and understanding study methods. Problems with comprehending consent contents could thus be irrespective of timing of the conversation.

A study by Janssen et al.^2^ considered most stroke patients incapable (as defined by self-formulated capacity rules) of providing consent prior to treatment due to neurological deficits. Approximately 20% of patients regained their ability to give consent within two days.^2^ Our results show that most patients considered themselves capable to give deferred consent even though the majority of the conversations happened within two days after stroke. Jansen et al.^17^ found that the capability of proxies to comprehend and recall the contents of informed consent in emergency settings could be impeded by emotional distress. Distress in the acute phase of stroke and uncertainty about the outcome of their loved one can render proxies less receptive to information, and less likely to consider trial participation.^17^ This was also mentioned by proxies in our study.

A general disapproval of the deferred consent method was reported by patients from the Endovascular Treatment for Small Core and Anterior Circulation Proximal Occlusion With Emphasis on Minimizing CT to Recanalization Times (ESCAPE) trial.^3,26^ However, respondents mostly agreed with the reasons given for the deferred consent procedure and none withdrew from the trial. A possible explanation for this finding may lay in the compared treatments: standard care alone was compared with additional EVT, the latter proved to be very beneficial. Patients do not always seem to grasp the concept of randomisation and may have felt that they were missing out on an effective treatment if allocated to the control arm. For the CONTRAST trials, intervention arm benefits may be less clear, and for most trials, the results are not yet known. Most respondents in ESCAPE did not comprehend the process of randomisation, similar to our study, suggesting an important misunderstanding on trial design, although previous studies showed that patients generally experience difficulties in understanding and remembering study methods and consent.^3,23,27^

### Strengths and limitations

We explored experiences of patients and proxies with deferred consent in acute stroke trials that are currently ongoing, as one of the first qualitative studies focusing on this topic. Theoretical sampling and iterative adjustment of the interview guide contribute to a heterogeneous sample reflecting opinions of varying patient groups. However, this study has limitations. Firstly, the overall positive result from the study may be exaggerated due to selection and consent bias, since we only interviewed participants who provided consent for the trial and agreed to participate in this study, which could have resulted in a sample with a relatively positive view on research and deferred consent. Patients and proxies often feel thankful when offered acute stroke treatment which could make them more likely to agree with study participation. However, only a very small proportion of patients refused consent,^5^ and we managed to include patients with both poor and good outcomes (or their proxies) in our study. Secondly, this study only included participants in the Netherlands, potentially limiting generalisability of our findings in other countries with cultural and geographical differences.^3^ Thirdly, interviews were conducted up to eleven months after consent. The elapsed time may play a role in memorisation and possible recall bias, although no large differences in memorisation were noted between patients with more or less elapsed time since giving consent.^3^ Fourth, we interviewed only 23 participants from a total population of 1305. Although we found that no additional subthemes could be identified from this point on, data from more trial patients could provide more robust results. Finally, if proxies gave consent, we mainly asked for their opinion, but additional information on patient’s experiences could have provided useful additional information.

Our findings can serve to support future studies investigating or using deferred consent. The current observations will be used as guidance to design a follow-up study among more CONTRAST trial patients. Notably, data on patients with severe aphasia and patients who refused participation would be valuable to create a more complete representation of patient and proxy opinions. Our study findings show room for improvement in patient recall, comprehension of study methods, understanding of the consent process and involvement of family members. Studying and development of methods to improve information transfer would be of great interest for future trials using deferred consent.

## Conclusion

Acute stroke trial participants included in our study usually considered deferred consent acceptable and felt capable of providing consent. In some cases, patients and proxies did not fully comprehend and recall the study methods explained in deferred consent conversations. The best timing for deferred consent remains unclear.

## Supplemental Material

sj-pdf-1-eso-10.1177_23969873211057421 – Supplemental Material for Patient and proxies’ attitudes towards deferred consent in randomised trials of acute treatment for stroke: A qualitative survey Click here for additional data file. Supplemental Material, sj-pdf-1-eso-10.1177_23969873211057421 for Patient and proxies’ attitudes towards deferred consent in randomised trials of acute treatment for stroke: A qualitative survey by Noa van den Bos, Sophie A van den Berg, Catalina MM Caupain, Jeannette AJ Pols, Tessa van Middelaar, Vicky Chalos, Diederik WJ Dippel, Yvo BWEM Roos, Manon Kappelhof and Paul J Nederkoorn in European Stroke Journal
