# Supplementary material for: Patient and proxies’ attitudes towards deferred consent in randomised trials of acute treatment for stroke: A qualitative survey
Source: Eur Stroke J. 2021 Nov 13;6(4):395–402. doi: 10.1177/23969873211057421 (PMC8948520; doi:10.1177/23969873211057421)
Supplement: sj-pdf-1-eso-10.1177_23969873211057421 – Supplemental Material for Patient and proxies’ attitudes towards deferred consent in randomised trials of acute treatment for stroke: A qualitative survey [file sj-pdf-1-eso-10.1177_23969873211057421.pdf]

APPENDIX I: INTERVIEW GUIDES

Including iteration details

Interview Guide – Patient

FINAL ADJUSTMENTS AFTER 20 INTERVIEWS (dd. 30-4-2020)

Text marked yellow is added compared to the first version (11-2019)

Text marked gray was deleted compared to the first version

For the interviewer:

The **bold questions** are meant as a clear guide. The rest of the questions serve as a possible tool or guidance.

Tips:

- Try to ask open questions as much as possible
- Let patients tell themselves about experiences / feelings as much as possible
  - Let the conversation flow
  - Discuss what the patient wants to discuss
- Focus on asking about deferred consent as much as possible
  - "Can you tell me something about giving permission for trial participation afterwards (after randomization)?"

QUESTIONNAIRE

When asking for situations: always ask for concrete examples. Preferably experiences, not hypotheses

Introduction

"A look back on the treatment and research"

Explain that the interviewer is independent / does not influence the treatment

Record

Ask for consent

Treatment (research comprehension, interpretation-focused)

"What happened?"

"Do you know anything about a trial/study that you took part in?"

"How come you do not remember this?"

If necessary: explanation about the trial. Hint: In [hospital] you were treated in a research setting.

"Can you tell a little more about this? (What is it about? What do they do?)"

"Do you know the purpose of the trial?" (why are there 2 options/randomisation)

"How were you informed about this trial?"

"What do you think of that?"

"**Which treatment did you receive?** Do you know why you received that treatment?"

"Such a study often requires a choice between two treatments. Was that also the case here? Why?"

If necessary, explanation about the treatment options in the trials.

"How was your treatment chosen?"

....

"How did you feel about your treatment being 'random'?"
"Did you experience it as a burden → cognitive/emotional"

<u>Conversation/Procedure</u> <u>(appreciation of Deferred Consent)</u>

When asking for situations: always ask for concrete examples. Preferably experiences, not hypotheses

And then you ended up in the hospital ward. A conversation took place, during which you were asked whether you wanted to give your permission to participate in a study/for the use of your data, and you had to sign something for this. We are going to talk about that now.

"How did this conversation to ask you for permission go?"
"How did you experience that conversation?"

....

"What actually happened, then?"

....

"How did you feel?"
"What did you give permission for?"

....

"What is the reason that it was only asked afterwards?"

....

"Can you tell me something about the condition you were in at the time of the interview? How did you feel?"

....

"Did you feel able to decide whether or not to participate in this study?"

....

"Why / What could that have to do with?"

....

"Who would you have preferred to have made the decision?" (e.g. physician/family)

....

"Would you have been okay with a family member making this decision for you?"

....

"And how do you feel about that now?"

....

"Why not?"

"Did you have enough time to decide?"

....

"Can you explain that / give an example?"

....

"Would you have preferred a different timing for the question to participate in the trial?"

....

"Which moment would you have preferred?"

....

"What is the reason for that?" "Why?"

....

"And what if they would have asked now? What do you think now?"

"What do you think about asking for permission in advance?" (better/worse)

"In what way would a different timing have affected your response?"

When asking for situations: always ask for concrete examples. Preferably experiences, not hypotheses

Final question: **"What do you think about asking consent for trial participation afterwards?** (So, asking patients for permission for their participation in a trial, after the treatment etc. has already taken place.)"

"Is this acceptable?"

"For what reason(s) do you think so?"

"On what does this depend for you?"

"Do you want to say, ask, or share anything else?"

Additional questions, in case of time left:

"Additional question: What is good and what could be improved, with regard to the process of asking consent?"

"Additional question: Would you have felt differently if you would have recovered differently from your stroke?"

"Can you explain that?"

"Additional question: How do you feel about research in general?"

LAATST AANGEPAST NA 20 INTERVIEWS (dd. 30-4-2020)
Geel gemarkeerde tekst is toegevoegd ten opzichte van de eerste versie (11-2019)
Grijs gemarkeerde tekst is verwijderd ten opzichte van de eerste versie

Voor de interviewer:
Let op! De **vetgedrukte vragen** zijn als duidelijke richtlijn. De rest van de vragen dienen als eventueel hulpmiddel of begeleiding.

Tips:

- Zoveel mogelijk proberen open vragen te stellen
- Zoveel mogelijk zelf laten vertellen over ervaring/gevoel
  - Laat het gesprek zijn vrije loop
  - Ga in op waar de patiënt op in wil gaan
- Zoveel mogelijk focussen op het achteraf toestemming vragen
  - "Kunt u iets vertellen over achteraf toestemming geven?"

VRAGENLIJST

Voorstellen
"Terugblikken op behandeling en onderzoek"
Vertellen dat de interviewer onafhankelijk is / geen invloed op behandeling heeft
Opname
Toestemming vragen

Behandeling (begrip onderzoek, interpretatie-gericht)

"Wat is er gebeurd?"

....

"Weet u iets over een onderzoek waar u aan meegedaan heeft?"

....

"Hoe kan het dat u zich dit niet herinnert?"

....

Eventueel uitleg over het onderzoek. Hint: In [ziekenhuis] hebben ze u behandeld in onderzoeksvorm.

"**Kunt u hier iets meer over vertellen**? (Waar gaat dat over? Wat doen ze?)"

....

"Weet u waar het onderzoek voor dient?" (waarom 2 opties/loten)

....

"Hoe bent u over dit onderzoek geïnformeerd?"

....

"Wat vindt u daarvan?"

....

"**Welke behandeling kreeg u**? Weet u waarom die?"

....

"Bij zo'n onderzoek moet vaak gekozen worden tussen 2 behandelingen. Was dat hier ook? Waarom?"

....

Eventueel uitleg over dat er meerdere behandelopties waren.

"Hoe werd gekozen welke behandeling u kreeg?"

Bij uitvragen van situaties: steeds concrete voorbeeldenvragen. Liefst ervaringen, geen hypotheses.

"Wat vond u ervan dat er werd geloot welke behandeling u kreeg?"
"Was het belastend → cognitief/emotioneel"

Gesprek/Procedure (waardering Deferred Consent)

En toen kwam u terecht op de afdeling. Er is u toen in een gesprek gevraagd of u toestemming wilde geven voor het deelnemen aan het onderzoek/gebruikmaken van uw gegevens en daarvoor moest u wat krabbels zetten. Daar gaan we het nu over hebben.

"Hoe verliep dit gesprek waarbij om toestemming werd gevraagd bij u?"
"Wat vond u van dit gesprek?"

"Wat gebeurde er dan eigenlijk?"

"Wat voelde u?"
"Waarvoor heeft u toestemming gegeven?"

"Wat is de reden dat het pas achteraf gevraagd werd?"

"Kunt u iets vertellen over de staat waarin u was op het moment van het gesprek? Hoe voelde u zich?"

"Voelde u zich in staat om te beslissen over deelname aan dit onderzoek?"

"Hoe kan dat / Waar zou dat mee te maken hebben?"

"Wie had u liever de beslissing willen laten maken?" (zoals arts/familie)

"Had u het toen goed gevonden als een familielid deze beslissing voor u had genomen?"

"En wat vindt u daar nu van?"

"Waarom niet?"

"Had u genoeg tijd om een beslissing te maken?"

"Kunt u dat toelichten / een voorbeeld geven?"

"Had u liever gewild dat de vraag voor deelname aan het onderzoek op een ander moment was gekomen?"

"Op welk moment had u dit liever gewild?"

"Wat is daar de reden voor?" "Waarom?"

"En wat als ze het nu vragen? Wat vindt u nu?"

Bij uitvragen van situaties: steeds concrete voorbeeldenvragen. Liefst ervaringen, geen hypotheses.

Bij uitvragen van situaties: steeds concrete voorbeeldenvragen. Liefst ervaringen, geen hypotheses.

"Wat vindt u van vooraf toestemming vragen?" (beter/slechter)

"Op welke manier zou de timing invloed gehad hebben op uw antwoord?"

Eindvraag: "Wat vindt u van achteraf toestemming vragen?" (Dus dat u pas achteraf wordt gevraagd om toestemming te geven - voor deelname aan dit onderzoek, nadat de behandeling e.d. al heeft plaatsgevonden.)"

"Is dit acceptabel?"

"Om welke reden(en) vindt u dat?"

"Waarvan is dit voor u afhankelijk?"

"Wilt u nog iets kwijt?"

Bonusvragen, te stellen bij tijd over:

"Bonus: Wat is er goed en wat kan er beter aan het proces van toestemming vragen?"

"Bonus: Had u er anders over gedacht als u er anders uit gekomen zou zijn?"

"Kunt u dat toelichten?"

"Bonus: Hoe staat u tegenover onderzoek in het algemeen?"

Interview Guide – Proxy

FINAL ADJUSTMENTS AFTER 20 INTERVIEWS (dd. 30-4-2020)
Text marked yellow is added compared to the first version (11-2019)
Text marked gray was deleted compared to the first version

For the interviewer:
The **bold questions** are meant as a clear guide. The rest of the questions serve as a possible tool or guidance.

Tips:

- Try to ask open questions as much as possible
- Let patients tell themselves about experiences / feelings as much as possible
  - Let the conversation flow
  - Discuss what the proxy wants to discuss
- Focus on asking about deferred consent as much as possible
  - "Can you tell me something about giving permission for trial participation afterwards (after randomization)?"

QUESTIONNAIRE

When asking for situations: always ask for concrete examples. Preferably experiences, not hypotheses

Introduction
"A look back on the treatment and research"
Explain that the interviewer is independent / does not influence the treatment
Record
Ask for consent

Treatment (research comprehension, interpretation-focused)

"What happened?"

....

"Do you know anything about a trial/study that your [*family member*] took part in?"

....

"How come you do not remember this?"

....

If necessary: explanation about the trial. Hint: In [hospital] your [*family member*] was treated in a research setting.

"Can you tell a little more about this? (What is it about? What do they do?)"

....

"Do you know the purpose of the trial?" (why are there 2 options/randomisation)

....

"How were you informed about this trial?"

....

"What do you think of that?"

....

"Which treatment did your [*family member*] receive? Do you know why your [*family member*] received that treatment?"

....

"Such a study often requires a choice between two treatments. Was that also the case here? Why?"

....

If necessary, explanation about the treatment options in the trials.

"**How was your [*family member's*] treatment chosen?"**

"How did you feel about your [*family* member's] treatment being 'random'?"
"Did you experience it as a burden → cognitive/emotional"

Conversation/Procedure (appreciation of Deferred Consent)

And then your [*family member*] ended up in the hospital ward. A conversation took place, during which you were asked whether you wanted to give your permission for your [*family member*] to participate in a study/for the use of your data, and you had to sign something for this. We are going to talk about that now.

"How did this conversation to ask you for permission go?"
"How did you experience that conversation?"

"What actually happened, then?"

"What did you give permission for?"

"What is the reason that it was only asked afterwards?"

"Can you tell me something about the condition you were in at the time of the interview? How did you feel?"

"Did you feel able to decide whether or not to participate in this study?"

"Why / What could that have to do with?"

"Who would you have preferred to have made the decision?" (e.g. physician/family)

"What do you think about that you had to decide as a [*family member*]?"

"Would you have been okay with another family member making this decision for your [*family member*]?"

"And how do you feel about that now?"

"Why not?"

"Did you have enough time to decide?"

"Can you explain that / give an example?"

"Would you have preferred a different timing for the question to participate in the trial?"

"Which moment would you have preferred?"

When asking for situations: always ask for concrete examples. Preferably experiences, not hypotheses

"What is the reason for that?" "Why?"

"And what if they would have asked now? What do you think now?"

"What do you think about asking for permission in advance?" (better/worse)

"In what way would a different timing have affected your response?"

"As a family member, you decided for your loved one. Which decision would your loved one have made?"

"What does that do to you?"

"What would you have done yourself?" (standard versus randomisation/experiment)

When asking for situations: always ask for concrete examples. Preferably experiences, not hypotheses

Final question: "What do you think about asking consent for trial participation afterwards? (So, asking patients for permission for their participation in a trial, after the treatment etc. has already taken place.)"

"Is this acceptable?"

"For what reason(s) do you think so?"

"On what does this depend for you?"

"Do you want to say, ask, or share anything else?"

Additional questions, in case of time left:

"Additional question: What is good and what could be improved, with regard to the process of asking consent?"

"Additional question: Would you have felt differently if your [*family member*] would have recovered differently from your stroke?"

"Can you explain that?"

"Additional question: How do you feel about research in general?"

LAATST AANGEPAST NA 20 INTERVIEWS (dd. 30-4-2020)
Geel gemarkeerde tekst is toegevoegd ten opzichte van de eerste versie (11-2019)
Grijs gemarkeerde tekst is verwijderd ten opzichte van de eerste versie

Voor de interviewer:
Let op! De **vetgedrukte vragen** zijn als duidelijke richtlijn. De rest van de vragen dienen als eventueel hulpmiddel of begeleiding.

Tips:

- Zoveel mogelijk proberen open vragen te stellen
- Zoveel mogelijk zelf laten vertellen over ervaring/gevoel
  - Laat het gesprek zijn vrije loop
  - Ga in op waar de vertegenwoordiger op in wil gaan
- Zoveel mogelijk focussen op het achteraf toestemming vragen
  - "Kunt u iets vertellen over achteraf toestemming geven?"

VRAGENLIJST

Voorstellen
"Terugblikken op behandeling en onderzoek"
Vertellen dat de interviewer onafhankelijk is / geen invloed op behandeling heeft
Opname
Toestemming vragen

Bij uitvragen van situaties: steeds concrete voorbeeldenvragen. Liefst ervaringen, geen hypotheses.

Behandeling (begrip onderzoek, interpretatie-gericht)

"Wat is er gebeurd?"

"Weet u iets over een onderzoek waar uw [*familielid*] aan meegedaan heeft?"

"Hoe kan het dat u zich dit niet herinnert?"

Eventueel uitleg over het onderzoek. Hint: In [ziekenhuis] hebben ze uw [*familielid*] behandeld in onderzoeksvorm.

"**Kunt u hier iets meer over vertellen**? (Waar gaat dat over? Wat doen ze?)"

"Weet u waar het onderzoek voor dient?" (waarom 2 opties/loten)

"Hoe bent u over dit onderzoek geïnformeerd?"

"Wat vindt u daarvan?"

"**Welke behandeling kreeg uw [*familielid*]**? Weet u waarom die?"

"Bij zo'n onderzoek moet vaak gekozen worden tussen 2 behandelingen. Was dat hier ook? Waarom?"

Eventueel uitleg over dat er meerdere behandelopties waren.

"**Hoe werd gekozen welke behandeling uw [*familielid*] kreeg?**"

"Wat vond u ervan dat er werd geloot welke behandeling uw [*familielid*] kreeg?"
"Was het belastend → cognitief/emotioneel"

Gesprek/Procedure (waardering Deferred Consent)

En toen kwam uw [*familielid*] terecht op de afdeling. Er is u toen in een gesprek gevraagd of u voor uw [*familielid*] toestemming wilde geven voor het deelnemen aan het onderzoek/gebruikmaken van uw gegevens en daarvoor moest u wat krabbels zetten. Daar gaan we het nu over hebben.

Bij uitvragen van situaties: steeds concrete voorbeeldenvragen. Liefst ervaringen, geen hypotheses.

"Hoe verliep dit gesprek waarbij om toestemming werd gevraagd bij u?"
"Hoe was dat voor u?"

"Wat vond u van dit gesprek?"

"Wat gebeurde er dan eigenlijk?"

"Waarvoor heeft u toestemming gegeven?"

"Wat is de reden dat het pas achteraf gevraagd werd?"

"Kunt u iets vertellen over de staat waarin u was op het moment van het gesprek? Hoe voelde u zich?"

"Voelde u zich in staat om te beslissen over deelname aan dit onderzoek?"

"Hoe kan dat / Waar zou dat mee te maken hebben?"

"Wie had u liever de beslissing willen laten maken?" (zoals arts/familie)

"Wat vindt u ervan dat u als [*familielid*] de beslissing heeft moeten maken?"

"Had u het toen goed gevonden als een ander familielid deze beslissing voor uw [*familielid*] had genomen?"

"En wat vindt u daar nu van?"

"Waarom niet?"

"Had u genoeg tijd om een beslissing te maken?"

"Kunt u dat toelichten / een voorbeeld geven?"

"Had u liever gewild dat de vraag voor deelname aan het onderzoek op een ander moment was gekomen?"

"Op welk moment had u dit liever gewild?"

"Wat is daar de reden voor?" "Waarom?"

"En wat als ze het nu vragen? Wat vindt u nu?"

"Wat vindt u van vooraf toestemming vragen?" (beter/slechter)

"Op welke manier zou de timing invloed gehad hebben op uw antwoord?"

"U heeft als familielid de beslissing voor uw naaste gemaakt. Welke beslissing zou uw naaste zelf hebben gemaakt?"

"Wat doet dat met u?"

"Wat zou u zelf hebben gedaan?" (standaard versus randomisatie/experimenteel)

Bij uitvragen van situaties: steeds concrete voorbeeldenvragen. Liefst ervaringen, geen hypotheses.

Eindvraag: "Wat vindt u van achteraf toestemming vragen? (Dus dat u pas achteraf wordt gevraagd om toestemming te geven - voor deelname aan dit onderzoek, nadat de behandeling e.d. al heeft plaatsgevonden.)"

"Is dit acceptabel?"

"Om welke reden(en) vindt u dat?"

"Waarvan is dit voor u afhankelijk?"

"Wilt u nog iets kwijt?"

Bonusvragen, te stellen bij tijd over:

"Bonus: Wat is er goed en wat kan er beter aan het proces van toestemming vragen?"

"Bonus: Had u er anders over gedacht als uw [*familielid*] er anders uit gekomen zou zijn?"

"Kunt u dat toelichten?"

"Bonus: Hoe staat u tegenover onderzoek in het algemeen?"

APPENDIX II: SUPPLEMENTAL FIGURES

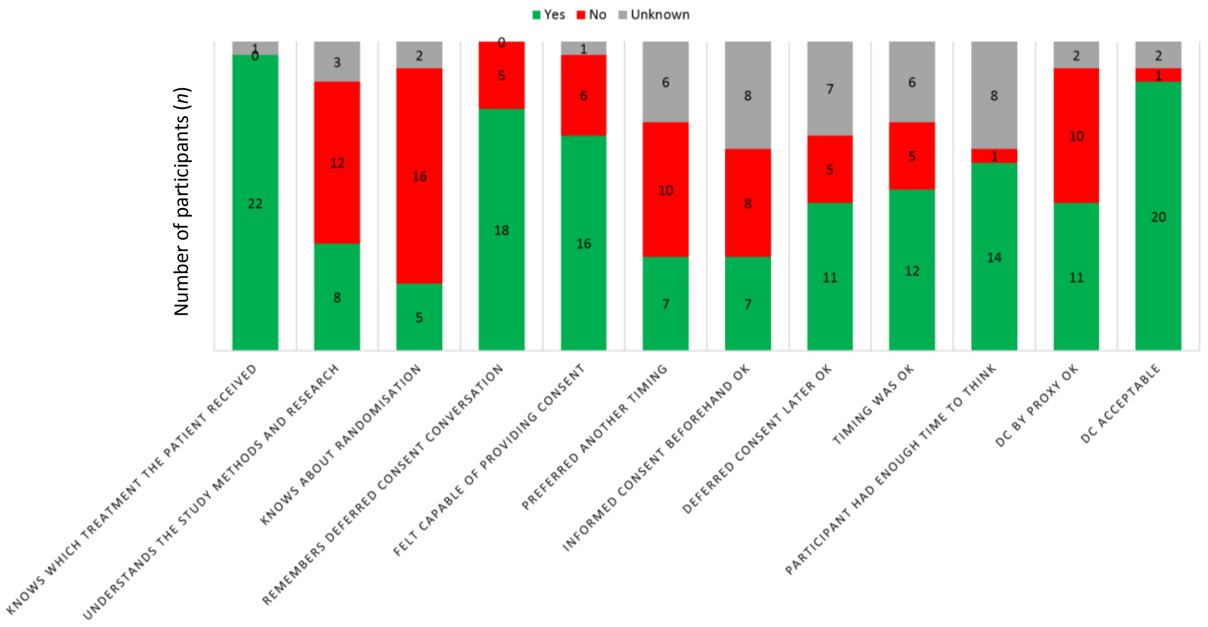

Figure S1: Overview of yes/no answers. n, number; OK, okay; DC, deferred consent. Study methods was defined as comparison of different treatment options.

APPENDIX III: SUPPLEMENTAL TABLES

Table S1: Identified Themes and Subthemes.

THEMES	RESEARCH AND STUDY METHODS	DEFERRED CONSENT
**SUBTHEMES**	Comprehension of the Trials<br>- Memory and Consciousness<br>- Setting<br>- Explanation<br>- Randomization<br>- Personal characteristics	Comprehension of the deferred consent procedure<br>- Memory and Consciousness<br>- Explanation, Language and Moderator<br>- Information forms
		Reasons for Consent<br>- Trust<br>- Interest and Knowledge<br>- Expectations and Experiences
		Feeling of capability<br>- Outcome and Expression<br>- Interest and Knowledge<br>- Support and Consultation<br>- Deferred consent by proxy and Autonomy
		Timing<br>- Beforehand or Deferred<br>- Time to Think<br>- Disclosure
		Acceptability<br>- Influencing Factors<br>- Consequences

Table S2: Quotations (English, translated)

Quotations

**Q1**	*No, I actually didn't even ask. Because I didn't even know that there were multiple treatments and why they chose it.* [P12]
**Q2**	*That was about that nitroglycerin patch, huh, that's what it was about. […] That is for research to, that when I get to the hospital, then you have already received pre-treatment. In the ambulance to, then it already widens the blood vessels, huh.* [P5]
**Q3**	*But, furthermore, I have not… It all happened and all in a panic at the beginning. And then a lot was said and, but well, that's all missing. I do not know all that.* [P14]
**Q4**	*Because I've also had three brothers with a stroke. So I know the clinical picture very well. And I used to be a nurse by profession as well, so I knew this and that about it.* [P13]
**Q5**	*So what I think afterwards, uh, why didn't they tell me exactly, uh, yeah, what they were going to do? Whether they thought I did not, in myself, or not, yes, how should I say that? Couldn't take it in at that moment. I actually didn't know exactly what they want to do. They may have thought: "She doesn't know what we're going to do anyway," but indeed, because I have a medical history, I did know it.* [P13]
**Q6**	*I am also thinking very carefully now, eh, because I can still picture having those conversations, but what they have told me exactly, I can't tell precisely. I know I gave permission for certain things, I remember that, or investigations, but which exactly, eh, I do not know.* [P10]
**Q7**	*Yes, every now and then they continued with all this medical abracadabra. Well, I thought: "It would be fine, though. He, standing here on the other side of the bed, because I don't know."* [P19]
**Q8**	*That was, a later paper said: "There were people who didn't get a patch and who did participate," […] then you could still participate and then, at a later date, you could still say that you dropped out, so. […] but I was in the hospital, so you have nothing else there, so uh… Then you can read that.* [P3]
**Q9**	*Because I also assume that they have expertise that I don't have, and that they have investigated things that I have not investigated and can't make a proper comparative assessment of then. So, with regard to that, I really rely on what the physicians recommend.* [P21]
**Q10**	*Yes, I know, it is a teaching hospital. And, uh, research is being done there. And I said: "And they learn from that, too." So, then I gave permission for it. […] I assumed that it uh, that it's always going that way.* [P12]
**Q11**	*But I hope the research actually brings it what the doctors uh, uh, provides insight into what is best for the patient. And that is actually the most important.* [P19]
**Q12**	*Yes, even before then. You say yes to something and you don't just do that. […] That is, I've signed that in full consciousness.* [P2]
**Q13**	*In my brain, I know everything. But I can't express… I can't express it. That was my problem. And it still is.* [P23]
**Q14**	*I always think I have a monopoly on wisdom, of course, but to decide in this field, you have to have some knowledgeability. And I don't have that.* [P6]
**Q15**	*But my daughter was there as well. And she works in the healthcare sector. She knows about everything and she had also read that and everything, so. And we discussed that first and she says: "That's fine." That's very pleasant. I've experienced a lot of ease with that.* [P3]

**Q16**	*My daughter did that, you know. […] (Daughter: And if you hadn't called, he wouldn't even have known about that research. […] And that was about the use of medicines and whether he wanted to participate in that.)* [P15]
**Q17**	*I guess that I, that you, especially if someone was in the same position as I was, shouldn't decide. Just not. I just think you should have someone who knows what he's talking about. Then you shouldn't tell someone, who is lying there, sick, so-and-so, no. They often make the wrong decisions. Yes, right? So, yes, no, I think so.* [P18]
**Q18**	*But on the other hand, yes, you're just in duty bound to do that. You don't think about that. He always took care of us, as I said, and then you just automatically do that in return.* [P7]
**Q19**	*But you will also forget things the longer you wait afterwards, so. I'm in favour of beforehand. But yeah. […] Yes, afterwards is afterwards, huh. Then there's actually no way back, sort of. […] If you know things in advance, you can say yes or no to that. And afterwards, I think: "Yes, oh, wait a minute."* [P8]
**Q20**	*I had enough time. Yes. Even though my decision was done immediately when I was talking to this person on the phone. [P1]*
**Q21**	*But I also don't think I was receptive to a lot of explanation at that time. At that moment.* [P13]
**Q22**	*What's done is done. Then you can't really go back, kind of. […] If you know things in advance, you can say yes or no. And when it's already done, that just makes me think 'yes, oh, wait a minute'.* [P8]
**Q23**	*Yes, that's why guys, and uh, at that point, action is needed, and you don't have time to explain for half an hour first, to explain something to, to depict how it all works. […] At such a moment, action is needed and we don't have time to quibble over rules first.* [P16]
**Q24**	*I think that for a research it does not make any difference how badly, if I had a stroke, then you always have some kind of deficiency, right. Uh, but… I do not know how badly it would affect me, but for a whole research, that does not make any difference, I think.* [P1]
**Q25**	*Because it doesn't do, uh, indirect damage, gosh, then who cares whether they applied that patch or not? […] So then, you know, it doesn't matter if you get it, have to give consent the next day.* [P3]

The quotes were translated from Dutch as literally as possible; possible grammatical mistakes due to aphasia were also maintained. Q, quotation; P, participant.

Table S3: Quotations (Dutch, original)

Quotations

**Q1**	*Nee, ik heb het eigenlijk niet eens gevraagd. Want ik wist niet eens dat er meerdere behandelingen er waren en waarom ze daarvoor gekozen hebben.* [P12]
**Q2**	*Ging over die nitroglycerine pleister, hè, daar ging het over. […] Dat is voor onderzoek om, dat als ik dan in het ziekenhuis komt, dan heb je al voorbehandeling gekregen. In de ziekenauto voor, dan verwijdt het al de bloedvaten, hè.* [P5]
**Q3**	*Maar verders heb ik daar ook niks van… Het is allemaal geleden en allemaal in paniek en al in het begin. En dan werd er veel gezegd en maar ja, da's allemaal zoek. Dat weet ik allemaal niet.* [P14]
**Q4**	*Omdat ik ook drie broers met een herseninfarct heb gehad. Dus ik ken het ziektebeeld heel erg goed. En ook, ik ben verpleegkundige van beroep geweest in d'r tijd, dus ik wist wel het één en het ander.* [P13]
**Q5**	*Wat ik dus achteraf wel eens denk, uh, waarom hebben ze me niet precies gezegd, uh, ja, wat ze gingen doen? Of ze toen dachten dat ik dat toch niet, in mezelf, of niet, ja, hoe moet ik dat zeggen? Niet op kon nemen op dat moment. Ik wist eigenlijk niet wat ze precies willen doen. Misschien hebben ze gedacht 'Ze weet toch niet wat we gaan doen,' maar inderdaad, omdat ik toch een medisch verleden heb, wist ik het dus wel.* [P13]
**Q6**	*Ik denk nu ook heel goed na, hè, want ik zie die beelden voor me, dat ik die gesprekken heb gehad, maar wat ze me nu precies hebben verteld, dat zou ik niet meer op het woord kunnen zeggen. Ik weet dat ik toestemming heb gegeven voor bepaalde dingen, dat weet ik nog wel of onderzoeken, maar welke precies, eh dat weet ik niet.* [P10]
**Q7**	*Ja, ze gingen af en toe zo met allemaal spreuken allemaal helemaal door. Nou, dat ik dacht van: 'Het zou wel goed zijn, hoor. Die hier aan de andere kant van het bed staat, want ik weet het niet.'* [P19]
**Q8**	*Dat was wel op een later papier stond: 'Er waren mensen die niet een pleister kregen en die wel meededen,' zeg maar. […] je kon dan nog meedoen en dan kon je op een later tijdstip nog zeggen dat je afhaakte, dus. […] maar ik lag in het ziekenhuis, dus je hebt niks anders daar, dus uh… Dan kun je dat lezen.* [P3]
**Q9**	*Want ik ga er ook vanuit dat zij expertise hebben die ik niet heb en dat zij dingen hebben onderzocht die ik niet heb onderzocht en dan geen goede afweging in kan maken. Dus ik vertrouw wat dat betreft wel heel erg op wat de artsen mij aanbevelen.* [P21]
**Q10**	*Ja, ik weet, het is een academisch ziekenhuis. Enne, uh, daar wordt onderzoeken in gedaan." En ik zei: "En daar leren ze ook van." Dus toen gaf ik toestemming ervoor. […] Ik ging er vanuit dat het uh, dat het altijd zo gaat.* [P12]
**Q11**	*Maar ik hoop dat het onderzoek eigenlijk het brengt wat de doctoren het uh, het uh, het inzicht brengt wat het beste is voor de patiënt. En dat is eigenlijk het voornaamste.* [P19]
**Q12**	*Ja, dat, ook al voor de tijd. Je zegt ja op iets en dat doe je niet zomaar. […] dat is met volle bewustzijn heb ik dat getekend.* [P2]
**Q13**	*In mijn hersenen weet ik alles. Maar ik kan het niet uit… Ik kan het niet uiten. Dat was mijn probleem. En dat heb ik nog steeds.* [P23]
**Q14**	*Ik denk wel altijd dat ik de wijsheid in pacht heb, natuurlijk, maar om de keuzes op dit vlak te maken, moet je toch wel enige kennis van zaken hebben. En die heb ik niet.* [P6]
**Q15**	*Maar daar was mijn dochter ook bij. En die zit in de zorg. Die weet van alles wel af en die had ook dat gelezen en alles,*

	dus. En daar had ik eerst mee overlegd en die zegt: "Dat is goed." Dat is heel prettig. Heel veel gemak van gehad. [P3]
**Q16**	*Dat heeft [naam dochter], mijn dochter heb dat gedaan, hoor. […] (Dochter: En al had u niet gebeld, dan had 'ie daar niet, dan had 'ie dat niet eens geweten van dat onderzoek. […] En dat ging over het gebruik van medicijnen en of dat 'ie daaraan mee wilde doen.)* [P15]
**Q17**	*Ik denk dat ik, dat je juist als iemand in de positie zat als ik was, geen keuze moet laten maken. Juist niet. Ik denk juist dat je dan iemand moet hebben die er verstand van heb. Dan moet je iemand niet die daar ziek ligt, zeggen van ja dit of zus, nee. Die maken vaak de verkeerde keuzes. Ja, toch? Dus, ja nee, dat vind ik.* [P18]
**Q18**	*Maar aan de andere kant, ja, dat doe je gewoon uit plicht. Daar denk je niet over na. Hij zorgde altijd voor ons, wat ik zei, en dan doe je dat gewoon automatisch terug.* [P7]
**Q19**	*Maar langer achteraf ga je ook weer dingen vergeten, dus. Ik ben wel een voorstander van vooraf. Maar ja. […] Ja, achteraf is achteraf, hè. Dan kan je eigenlijk niet meer terug, soort van. […] Als je dingen van tevoren weet, kan je daar ja of nee op zeggen. En achteraf denk ik 'Ja, oh, wacht effe.'* [P8]
**Q20**	*I had enough time. Ja. Even though my decision was done immediately when I was talking to this person on the phone.* [P1]
**Q21**	*Maar ik geloof ook niet dat ik ontvankelijk was voor heel veel uitleg op dat moment. Op dat moment.* [P13]
**Q22**	*Achteraf is achteraf. Dan kan je eigenlijk niet meer terug, soort van. […] Als je dingen van tevoren weet, kan je daar ja of nee op zeggen. En achteraf denk ik 'Ja, oh, wacht effe.'* [P8]
**Q23**	*Ja, daarom jongens, en uh, op dat moment moet er gehandeld worden en heb je geen tijd om eerst eens een half uur nog uit te leggen, wat uit te leggen naar uit te tekenen hoe dat allemaal in mekaar zit. […] Op zo'n moment moet er gewoon gehandeld worden en dan mogen niet eerst allemaal een beetje gaan zuchten over regeltjes.* [P16]
**Q24**	*I think that for a research it does not make any difference how badly, if I had a stroke, then you always have some kind of deficiency, right. Uh, but… I do not know how badly it would affect me, but for a whole research, that does not make any difference, I think.* [P1]
**Q25**	*Omdat het geen uh, indirecte schade aanricht, goh, wat maakt het ons dan uit of die pleister geplakt is of niet? [...] Dus dan, weet je, dan maakt het niet uit of je de volgende dag krijgt, toestemming moet geven.* [P3]

Q, quotation; P, participant.

Table S4: Quotations concerning the best timing of Deferred Consent

TIMING OF DC	OPINION (+/-)	QUOTATION
**Beforehand (Informed Consent)**	+	*Yes, just if you know, just say it. You don't have to wait for me to calm down or something. The moment you tell me something, then you think, I can sit and listen quietly […] I don't care what time, but rather immediately. If something has to be done, there were all kind of things that had to happen, rather immediately.* [P10]
	-	*It is all very confusing, and you have people who panic and then you just sort of, uh, and then you bother them with these kind of questions when they do not know whether they will live or be paralysed or not, you know, it is not… I do not think it is a good place to do it. Or time.* [P1]
**Afterwards (Deferred Consent)**	+	*I didn't find it that appropriate, beforehand. I mean, I still was very ill, and yes, not thinking as clearly as I would say. So I would always do it afterwards.* [P13]
	-	*No, because then [beforehand], I think, it would have been of little use. If they ask afterwards, you think, yes, that's not good either.* [P2]
- **Day 0-1 (DC)**	+	*No, I do, that as soon as possible, uh… Discuss it and report what's going on. Because, I suppose, if I say I want to have [another treatment*], I don't want to participate, that they might have given that.* [P22]
	-	*But when you're in an emotional rollercoaster every time when someone is admitted to the hospital and you have to go there several times a day or every day, then, I can imagine you think uh, if someone approaches you for research, 'Well, never mind.'* [P7]
- **Day 2-5 (DC)**	+	*It wasn't necessary to do it earlier, and at a later time, you're more used to your situation. You're used to what you've got, what they've done, uh, you know here's where I'm going to stay for now. And then you can come to terms with that. So. I think it's a good thing that there's some time in between, before they come up with, uh, with their questions. So that you can process what has happened.* [P12]
	-	*But of course, my father could have died and then you have to, if they do it a day or two afterwards, you'll be confronted with the patient's death and you will still have to ask for permission. I don't think that will be very useful for a neurologist.* [P4]
- **Week 2-4 (DC)**	+	*And the first week, second week, you don't know. Because everything in your life is one big blur. Yes, I'm saying it right. Just a blur, that they just ask permission and well, it must be fine and it is yes and no, and, but three weeks later or two weeks later you know what you give permission for.* [P19]
- **>2 months (DC)**	+	*And there has been so much excitement. From uh, from [place name] to [place name], from [place name] to [place name] and from [place name] to rehabilitation. There she suffered from mild corona. Then she had to be locked up, no visitors were allowed anymore. It has been nothing for eight weeks*. [P14]

	-	*Right now, I still wouldn't have known, I guess. No, I know… because if I still remember yesterday, I've already forgotten some things. I can't remember.* [P15]
**Does not matter**	+	*I'm someone who finds it very important. So that they, yes, have enough information to be able to treat people in the right and most effective way in the future. So I would always have given permission. Whether it is immediately after or three days after treatment. That makes, it wouldn't have made a difference to me. Maybe to other people, but not to me.* [P13]

The quotes were translated from Dutch as literally as possible; possible grammatical mistakes due to aphasia were also maintained.

*Received treatment was masked to preserve blinding of allocated treatment. DC, deferred consent; +, positive view of the mentioned timing; -, negative view of the mentioned timing; P, participant.

Table S5: Quotations concerning Acceptability of Deferred Consent: Influencing Factors

FACTOR	INFLUENCE (+/-)	QUOTATION
**(Emergency) setting**	+	*I consider this to be an emergency. That's how I see it. And yes, then, yes, then the patient takes precedence over any questions they may have. [...] But normally it would be better if they asked such a question in advance.* [P12]
**Outcome**	+	*I have no problem with that. I've been treated well, that's most important*. [P11]
	-	*If that treatment had not been successful and she would have been completely in need of help and knew nothing about it, then it would raise some questions, I think.* [P16]
**Risks and burden**	+	*It is just a matter of fact, it has happened, are you willing to… or not, to after… further… to indicate and then it's just... physically it has nothing to do with it, just a conversation and questions.* [P23]
	-	*In my opinion, we shouldn't have done that. All the drama. We can't keep moving her around. I also indicated that later. [...] And my mom also doesn't feel like it at all, all those, uh, all those antics.* [P8]
**Treatment**	+	*The treatment differed, diff… diff… it would diff… or the treatment didn't change. The only thing was that there could be a conversation, uh… following this period.* [P23]
	-	*If my father needs surgery and I get to hear it afterwards, then I'm fine with it as well. But it shouldn't be that they have amputated his leg or whatever, to see if uh and that he is missing his leg.* [P10]
**Research purposes**	+	*I just think: 'If it's better and they can do something with it in the future, that I, yes, that they can get a better treatment because of that, or whatever, yes, then I'm okay with it.'* [P9]
	-	*Well, I was wondering here and there: 'What's the point of it?' I've already had surgery, you know. And then signing and having to read things, which didn't go very well at all.* [P6]
**Lack of awareness**	+	*Consent afterwards. Yes… I think that's acceptable, because I, I think if you have to do it afterwards, then you know what you give permission for.* [P19]
	-	*Afterwards, I was asked to participate in a study. Anyway, at that point, they already made their choice. So, you participate in a study on which you actually have little, no influence*. [P22]

The quotes were translated from Dutch as literally as possible; possible grammatical mistakes due to aphasia were also maintained. +, beneficial contribution to the deferred consent acceptance; -, adverse contribution to the deferred consent acceptance. P, participant.

Table S6: Yes or No? An overview of the answers.

	1	2	3	4	5	6	7	8	9	10	11	12	13	14	15	16	17	18	19	20[$]	21	22	23
**Knows which treatment the patient received**																	-						
**Understands study methods**							-										-						-
**Knows about randomization**																	-				-		
**Remembers DC conversation**																				X			
**Felt capable of providing DC**															#	-				X			
**Preferred another timing of DC**				-					-	-	-					-				X			-
**Informed consent beforehand OK**		*			-	-					-			-	-		-	-		-			
**DC later OK**	-				-	-			-		-					-		-					
**Current timing of DC OK**						-			-		-			-	-	-							
**Enough time for reflection**					-			-						-	-	-	-			X	-		
**DC by proxy OK**				* *			* *	* *	* *	* *				* *	#	-	-						

**DC acceptable**														-	D		-						

This table shows the answers of participants (1-23) to certain questions. Green means ‘yes’, red is ‘no’. *This patient would not want to sign the deferred consent form in advance, but would like to be informed and give verbal informed consent before treatment. **Proxies were asked if they preferred someone else to sign. #, according to daughter who answered due to patient’s aphasia. $, patient 20 had only received explanation and provided verbal consent at the time of the interview and signed the papers two months later. *Abbreviations: DC, deferred consent; OK, okay; -, unknown or not discussed; X, not applicable.*

APPENDIX IV: LIST OF CONTRAST CLINICAL TRIAL COLLABORATORS

Research leaders

Diederik Dippel (MD, PhD),[2] Charles Majoie (MD, PhD)[1]

Consortium coordinator:

Rick van Nuland (PhD)[3]

Imaging assessment committee

Charles Majoie (MD, PhD) – *Chair*,[1] (Amsterdam Medical Center, location AMC); Aad van der Lugt (MD, PhD) – *Chair*,[2] Wim van Zwam (MD, PhD),[4] Linda Jacobi (MD, PhD),[4] René van den Berg, (MD, PhD),[1] Ludo Beenen (MD),[1] Bart Emmer (MD, PhD),[1] Adriaan van Es, (MD, PhD),[2] Pieter-Jan van Doormaal (MD),[2] Geert Lycklama (MD, PhD),[7] Ido van den Wijngaard (MD, PhD),[7] Albert Yoo (MD, PhD),[19] Lonneke Yo (MD, PhD),[6] Jasper Martens (MD, PhD),[8] Bas Hammer (MD, PhD)[11], Stefan Roosendaal (MD, PhD),[2] Anton Meijer (MD, PhD),[10] Menno Krietemeijer (MD)[6], Reinoud Bokkers (MD, PhD)[16], Anouk van der Hoorn (MD, PhD)[16], Dick Gerrits (MD)[13]

Adverse event committee

Robert van Oostenbrugge (MD, PhD) – *Chair*,[4] Bart Emmer (MD, PhD),[2] Jonathan Coutinho (MD, PhD),[1] Ben Jansen (MD, PhD)[17]

Outcome assessment committee

Yvo Roos (MD, PhD) – *Chair*,[1] Sanne Manschot (MD, PhD),[7] Diederik Dippel (MD, PhD),[2]

Henk Kerkhof (MD, PhD),[15] Ido van den Wijngaard (MD, PhD),[7] Jonathan Coutinho (MD, PhD),[1] Peter Koudstaal (MD, PhD),[2] Koos Keizer (MD, PhD)[6], Jelis Boiten (MD, PhD)[7]

Data management group

Hester Lingsma (PhD),[2] Diederik Dippel (MD, PhD)[2], Vicky Chalos (MD),[2] Olvert Berkhemer (MD, PhD)[2]

Imaging data management

Aad van der Lugt (MD, PhD),[2] Charles Majoie (MD, PhD),[1] Adriaan Versteeg,[2] Lennard Wolff (MD),[2] Jiahang Su (MSc)[2], Manon Tolhuisen (MSc)[1], Henk van Voorst (MD)[1]

Biomaterials and translational group

Hugo ten Cate (MD, PhD),[4] Moniek de Maat (PhD)[2], Samantha Donse-Donkel (MD),[2] Heleen van Beusekom (PhD),[2] Aladdin Taha (MD)[2]

Local collaborators

Vicky Chalos (MD),[2] Kilian Treurniet (MD),[1] Sophie van den Berg (MD),[1] Natalie LeCouffe (MD),[1] Rob van de Graaf (MD),[2] Robert-Jan Goldhoorn (MD),[4] Aladdin Taha (MD),[2] Samantha Donse-Donkel (MD),[2] Wouter Hinsenveld (MD),[4] Anne Pirson (MD),[4] Lotte Sondag (MD),[10] Manon Kappelhof (MD),[1] Rik Reinink (MD),[5] Manon Tolhuisen (MD),[1] Josje Brouwer (MD),[1] Lennard Wolff (MD),[2] Sabine Collette (MD),[16] Irati Schonewille,[9] Wouter van der Steen (MD)[2], Simone Uniken Venema (MD)[5]

Research nurses

Rita Sprengers,[1] Martin Sterrenberg,[2] Naziha El Ghannouti,[2] Sabrina Verheesen,[4] Jasmijn Lodico,[13] Hanneke Droste,[13] Wilma Pellikaan,[9] Kitty Blauwendraat,[9] Yvonne Drabbe,[11] Joke de Meris,[7] Michelle Simons,[8] Hester Bongenaar,[6] Anja van Loon,[14] Karin Kraus,[14] Eva Ponjee,[12] Rieke Eilander,[12] Suze

Kooij,[15] Marieke de Jong,[16] Esther Santegoets,[17] Annemarie Slotboom[15], Ayla van Ahee,[1,5] Marinette Moynier,[18] Annemie Devroye,[19] Evelyn Marcelis,[19] Ingrid Iezzi,[20] Annie David[20]

Study monitors

Leontien Heiligers[2], Yvonne Martens[2]

Affiliations

[1] Amsterdam UMC, University of Amsterdam, Amsterdam, the Netherlands;

[2] Erasmus MC University Medical Center, Rotterdam, the Netherlands;

[3] Lygature, Utrecht, the Netherlands;

[4] Cardiovascular Research Institute Maastricht (CARIM), Maastricht University Medical Center, Maastricht, The Netherlands;

[5] University Medical Center Utrecht, Brain Center, Utrecht, the Netherlands;

[6] Catharina Hospital, Eindhoven, the Netherlands;

[7] Haaglanden Medical Center, the Hague, the Netherlands;

[8] Rijnstate Hospital, Arnhem, the Netherlands;

[9] St. Antonius Hospital, Nieuwegein, the Netherlands;

[10] Donders Institute for Brain, Cognition and Behaviour, Centre for Neuroscience, Radboud University medical center, Nijmegen, The Netherlands;

[11] HagaZiekenhuis, the Hague, the Netherlands;

[12] Isala Klinieken, Zwolle, the Netherlands;

[13] Medisch Spectrum Twente, Enschede, the Netherlands;

[14] Amphia Hospital, Breda, the Netherlands;

[15] Albert Schweitzer Hospital, Dordrecht, the Netherlands;

[16] University Medical Center Groningen, the Netherlands;

[17] Elisabeth-TweeSteden Hospital, Tilburg, the Netherlands;

[18] Centre Hospitalier Universitaire de Montpellier, Montpellier, France;

[19] Universitair Ziekenhuis Leuven, Leuven, Belgium;

[20] Centre Hospitalier Chrétien, Liège, Belgium;

[21] National Hospital for Neurology and Neurosurgery, London, United Kingdom;

[22] Institute of Neuroscience and Newcastle University Institute for Ageing, Newcastle University, Newcastle, United Kingdom;

[23] London School of Hygiene & Tropical Medicine, London, United Kingdom;

[24] Texas Stroke Institute, Plano, Texas, United States of America
